# Supplementary material for: Crystal structure of an invertebrate cytolysin pore reveals unique properties and mechanism of assembly
Source: Nat Commun. 2016 May 12;7:11598. doi: 10.1038/ncomms11598 (PMC4865846; doi:10.1038/ncomms11598)
Supplement: Supplementary Information — Supplementary Figures 1 - 7 and Supplementary References 1 - 3 [file ncomms11598-s1.pdf]

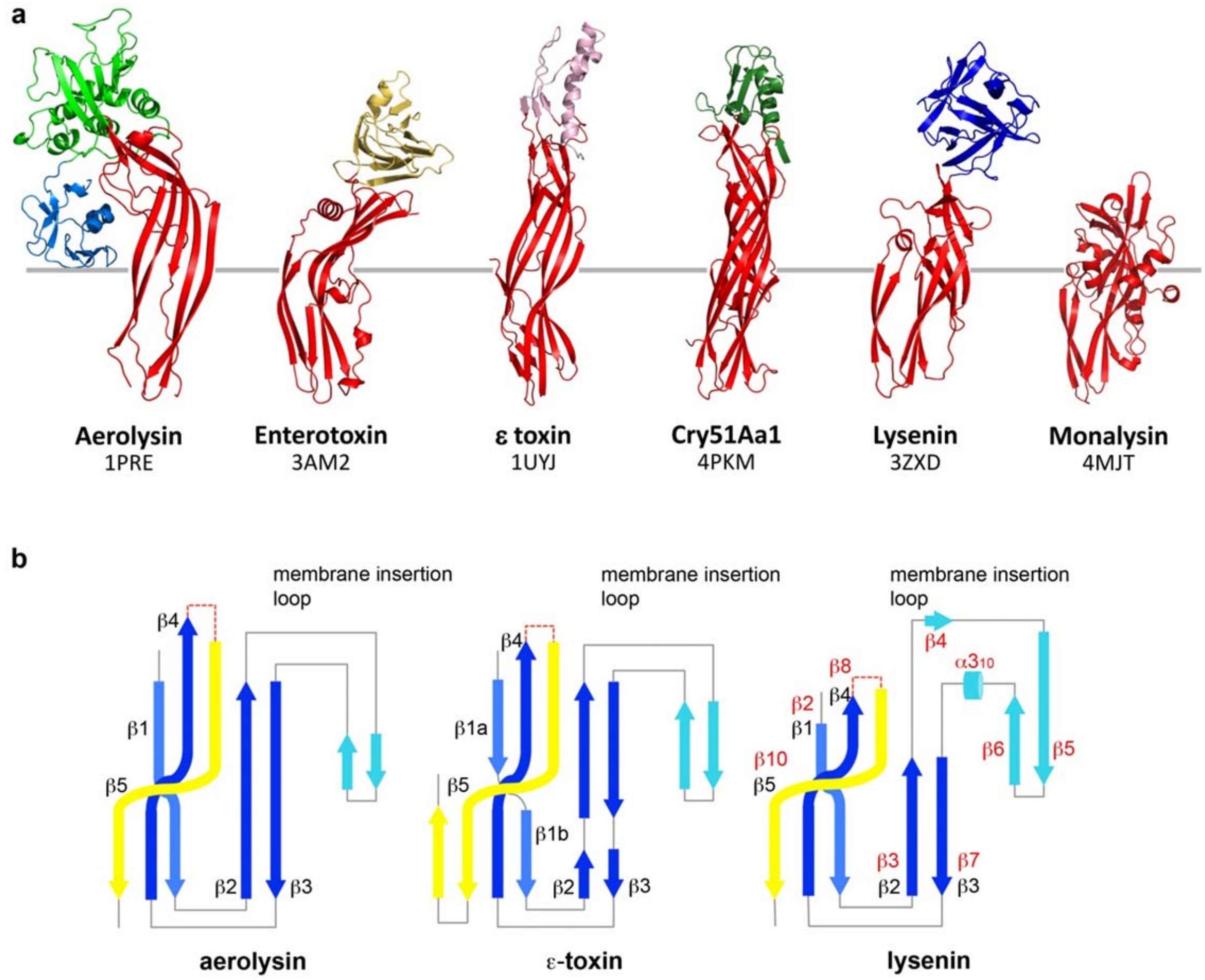

Supplementary Figure 1 │ Structures of some representatives of aβ-PFTs.. (**a**) The β-strand rich pore-forming module (PFM), which is a conserved evolutionary feature[1], is coloured red. Other domains that are present in different representatives are coloured differently. Most aβ-PFTs contain only one additional domain, which is generally used for attachment to cellular membranes. Aerolysin is an exception, since it contains two additional domains. Monalysin on the other hand does not contain any additional domains, but some additional residues within its PFM. Aerolysin contains also a C-terminal peptide, which is required for folding and must be proteolytically removed in order to allow the oligomerization (not shown here). The PFM is usually divided in two subdomains, here delimited by a grey line. Aerolysin forms pores out of seven protomers, while lysenin and monalysin form pores by assembling nine protomers. A model of the pore structure exists for aerolysin[2], but no high-resolution structural information about pores is available for any member of the aβ-PFTs family, apart from lysenin (this work). (**b**) Topological diagram of the PFM of three representatives of aβ-PFTs based on a common core topology of the pore-forming module suggested by Szczesny et al[1]. Blue, core with conserved sequence; red, variable loop (marked as a dashed red line, indicating various sizes of these regions in different structures); yellow: weekly conserved β-strand. Beta strands are numbered according to the consensus from Szczesny et al[1]. For lysenin, numbering of secondary structure elements in red follows the numbering in Fig 4a.

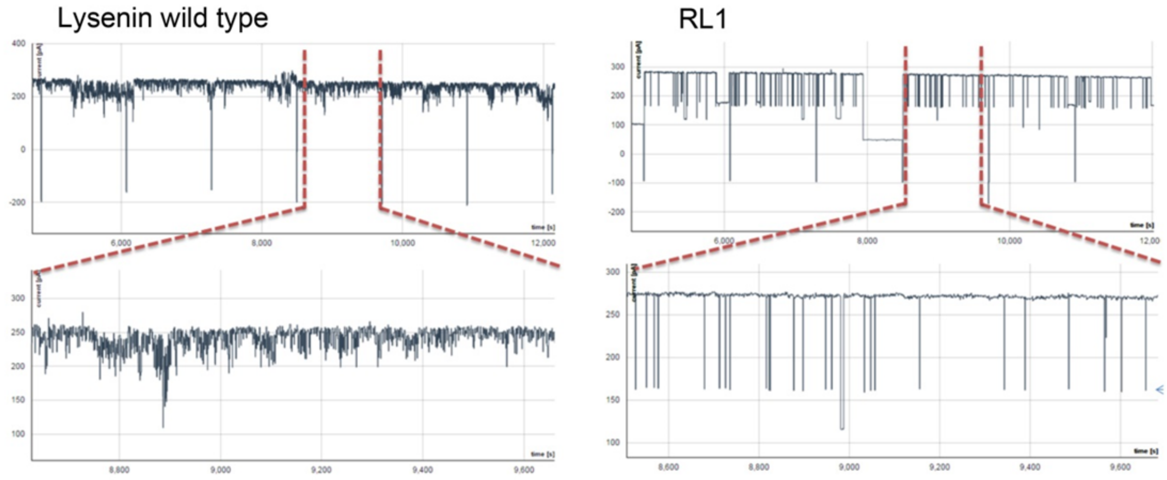

Supplementary Figure 2 │ Translocation of DNA through RL1 pore. (a) Single channel current recordings of the wild-type lysenin and RL1 pores. A noisy open pore current of approximately 250 pA is observed with no evidence of DNA translocation in the case of the wild-type lysenin. A quieter open pore current of approximately 275 pA is observed for RL1. Short downward spikes (160 pA blockings) are caused by the thrombin binding aptamer translocating through the pore.

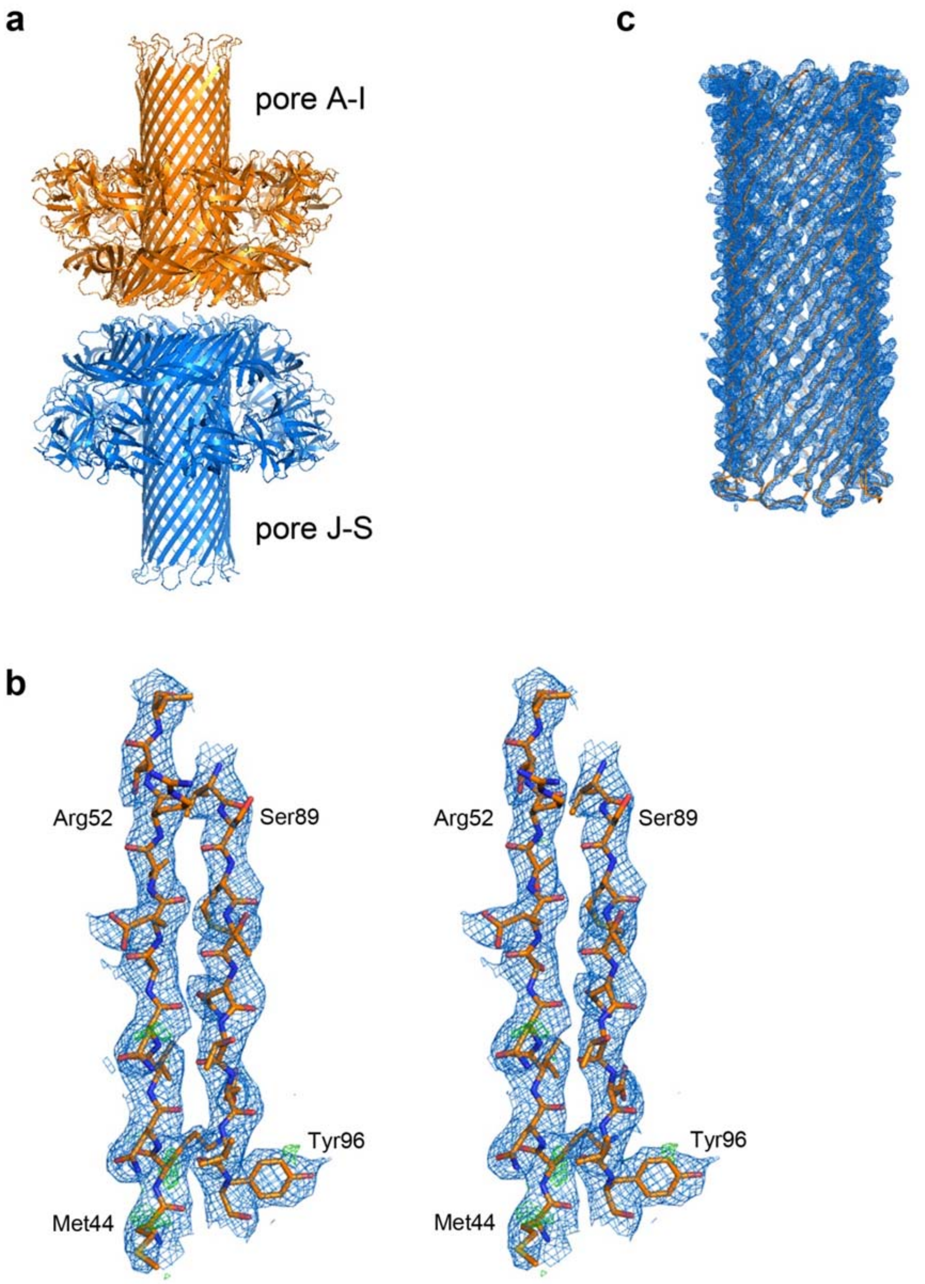

Supplementary Figure 3 │Crystallographic features of the lysenin pore. (**a**) Two nonameric oligomers were present in the asymmetric unit, packed in a head-to-head manner Ribbon representation: pore with protomer chains A-I is in orange and the pore with chains J-S in blue. For a clearer view, all loops on the trans side of the pore are depicted. Every protomeric subunit of the two pores is completely defined, except for 9 N-terminal residues, and the loops connecting the β-hairpins at the *trans* side of the barrel, that could be traced in only 2 protomers in one (chains A-I) and 6 in the other (chains J-S) pore. (**b**) 2Fo-Fc (contoured at 1 σ) electron density map (blue mesh) and Fo-Fc electron density map (contoured at 2.5 σ) (green mesh) of the region in the β-barrel, shown in stereo view. Stick representation (carbon: orange, oxygen: red, nitrogen: blue, sulphur: yellow). (**c**) 2Fo-Fc (contoured at 1 σ) electron density map (blue mesh) along the β-barrel. For clarity, only Cα atoms are shown (orange skeleton) in the electron density.

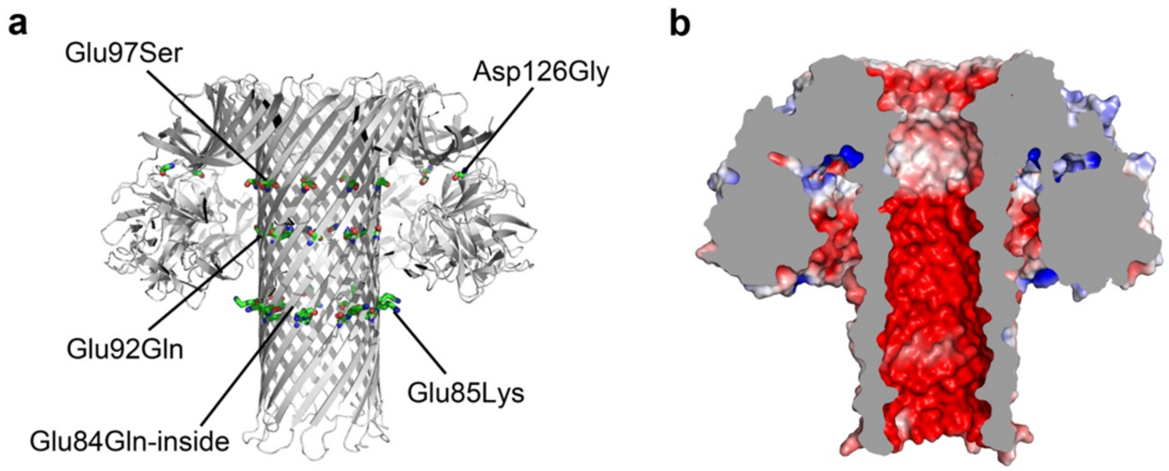

Supplementary Figure 4 │ Mutations in RL1 and properties of lysenin pore. (**a**) Position of five mutations in RL1 (left). Ribbon representation of the pore (grey). Amino acids that were mutated are presented as sticks (carbon: green, oxygen: red, nitrogen: blue). (**b**) The electrostatic properties of the inner surface of the model of the wild-type lysenin pore (right). The surface of the pore is colored according to electrostatic potential. Cut off -5 kT/e was used for the negative potential (red) and +5 kT/e for the positive potential (blue).

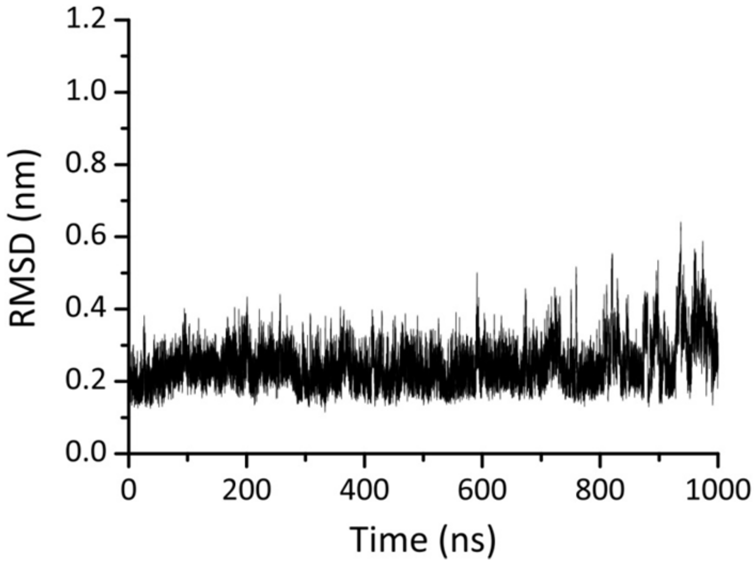

Supplementary Figure 5 │ Flexibility of the lysenin monomer. RMSD of lysenin backbone over 1µs. Fluctuations occur mainly because of the flexibility between N- and C-terminal domains, in agreement with crystallographic data[3].

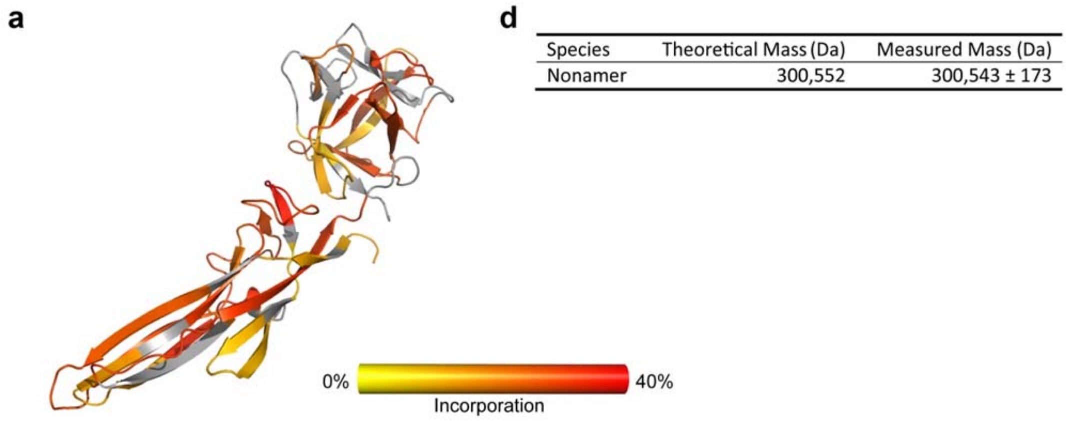

d

Species	Theoretical Mass (Da)	Measured Mass (Da)
Nonamer	300,552	300,543 ± 173

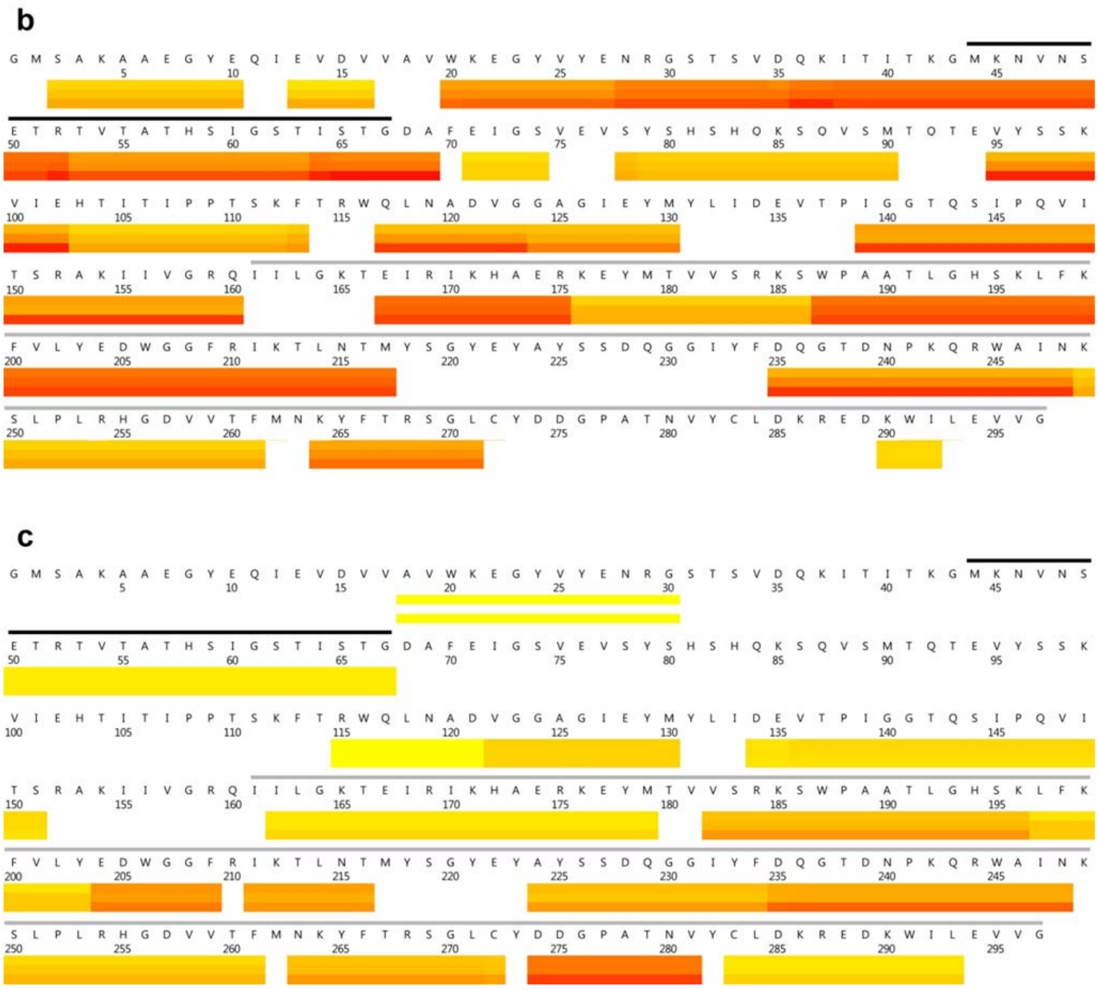

Supplementary Figure 6 │ Relative solution exposure of lysenin. HDX was used to measure the relative solution exposure of lysenin in both monomeric and pore form. (**a**) The relative fraction of deuterium uptake mapped to the monomeric form of the lysenin (PDB-ID 3ZXD). (**b**) Heat map representation of the monomer deuterium uptake at 20 s, 100 s and 1000 s, aligned to its sequence. **c**, Heat map sequence representation of deuterium uptake of the pore at 20 s, 100 s, 1000 s. Amino acid sequence of the lysenin construct used in our study is shown. The black line in **b** and **c** marks the tongue region and the grey line marks the C-terminal domain. Residue number is marked according to the lysenin sequence as present in the crystal structure. (**d**) Measured and theoretical mass of the lysenin nonamer from non-denaturing mass spectrometry.

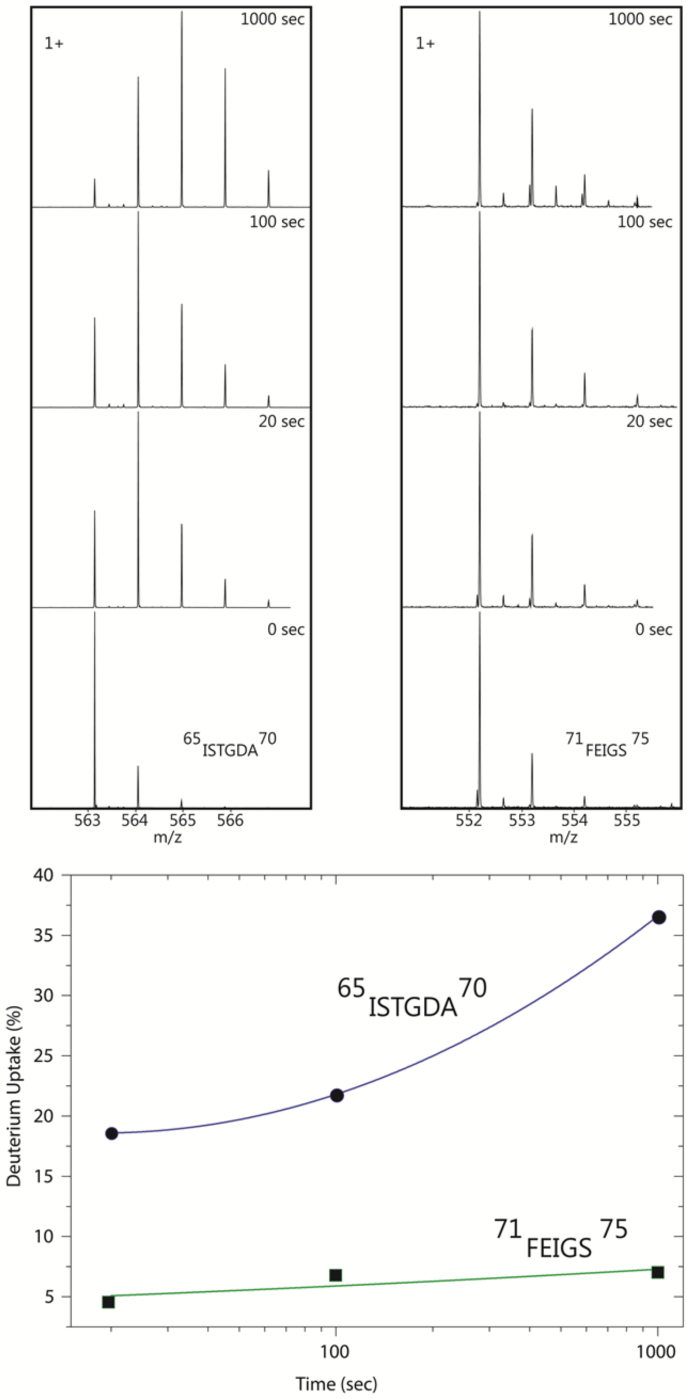

Supplementary Figure 7 │ Mass spectra and deuterium uptake summary for two selected peptides in the monomer lysenin. The peptide ISTGDA is in the tongue region of the protein and shows relatively high deuterium uptake compared to the peptide (FEIGS) from the adjacent protein region.

References

1. Szczesny, P., Iacovache, I., Muszewska, A., Ginalski, K., van der Goot, F.G. & Grynberg, M. Extending the aerolysin family: from bacteria to vertebrates. *PLoS ONE* **6**, e20349 (2011).

2. Degiacomi, M.T., *et al.* Molecular assembly of the aerolysin pore reveals a swirling membrane-insertion mechanism. *Nature Chem. Biol.* **9**, 623-629 (2013).

3. De Colibus, L., *et al.* Structures of lysenin reveal a shared evolutionary origin for pore-forming proteins and its mode of sphingomyelin recognition. *Structure* **20**, 1498-1507 (2012).
